# Editorial: Mechanisms of resistance to the targeted therapy and immunotherapy in cutaneous melanoma

**DOI:** 10.3389/fonc.2022.1016901

**Published:** 2022-08-29

**Authors:** Sanjay Premi, Yong Qin, Nihal Ahmad

**Affiliations:** ^1^ Department of Tumor Biology, Moffitt Cancer Center, Tampa, FL, United States; ^2^ School of Pharmacy, University of Texas at El Paso, El Paso, TX, United States; ^3^ Department of Dermatology, University of Wisconsin, Madison, WI, United States

**Keywords:** cutaneous melanoma, immunotherapy, targeted therapy, drug resistance, editorial

Cutaneous melanoma, the cancer of pigmented melanocytes, is one of the most aggressive skin cancers (1). Targeted inhibitors and immunotherapies have substantially improved the overall survival rate of patients with metastatic melanoma. However, acquired resistance is often inevitable in addition to the side effects or lack of clinical effects, especially for immunotherapy. Based on recent studies, several mechanisms have been elucidated so far, which describe the development of resistance to kinase inhibitors. The most common of them are re-activation of the MAPK pathways and sustained ERK activity (2) or activation of alternate pathways (3, 4), which allow melanoma cells to tolerate and get addicted to the targeted therapies. In addition, there are alterations in the tumor microenvironment, phenotype switching, and selection of resistant cells by the targeted therapeutics. Similarly, even after initial success, immunotherapies like T-cell checkpoint blockade may become ultimately ineffective owing to overexpression of alternate checkpoints (5), ineffective antigen presentation machinery (6), or loss of interferon response.

Despite ample advances in targeted therapy (BRAFi, MEKi) and immunotherapies (checkpoint inhibitors), cutaneous melanoma still shows a poor prognosis due to the development of intrinsic and/or acquired resistance. Identification of novel pro-tumorigenic, proliferative, and metastatic mechanisms is needed so that new therapeutic targets can be identified. In the current issue, the studies have described and discussed several such mechanisms which could be utilized as novel vulnerabilities against melanoma.

Han et al. described the possible functions of “minichromosome maintenance protein complex (MCM) as a prognostic marker in cutaneous melanoma. Using bioinformatics and biostatistics, the authors described the upregulation of MCMs in melanoma, their association with cancer progression and stages, and their effects on overall melanoma survival. Additionally, a correlation between differential expression of MCMs and tumor immune cell infiltration was described. The authors concluded that while MCMs are excellent prognostic markers, the underlying mechanisms could be explored to better understand their molecular functions in melanoma.

Autophagy is an inherent mechanism of clearing out damaged cells for the regeneration of healthier cells, utilized by the cancer cells for their survival and induction of therapeutic resistance. Degan et al. described a tentative combination of the autophagy inhibitor, chloroquine (CQ), with targeted MEKi, Trametinib, in inhibiting melanoma cell proliferation. The authors described an enhanced therapeutic effect using this combination in comparison to the single agent. Reduced cell proliferation was attributed to the decreased ERK activity by the combination, which remained almost unaffected by CQ alone. However, the reduction of tumor infiltration by the CD4 and CD+8 T lymphocytes in response to the CQ + MEKi combination limits its clinical usage. The authors suggest designing unique drug regimes to suppress the detrimental effects of CQ and enhance the ratio of benefits over risks of combining CQ with other therapeutic drugs.

Immunotherapy, especially checkpoint inhibitors, has revolutionized the therapeutic approaches against melanoma. However, ultimately, patients develop resistance. In this regard,  Thornton et al. reviewed the available immunotherapeutic approaches in detail, which includes immune checkpoint inhibitors (anti-PD-1/anti-CTLA-4 ICIs) and oncolytic enhanced immunotherapy. In addition, the authors briefly described tumor mutation burden, anti-immune signaling in the tumors, and epigenetic regulators as possible mechanisms of resistance against immunotherapy. Interestingly, this review article discussed the inhibition of two or more immune checkpoints simultaneously or sequentially. Since the resistance mechanisms are unclear, detailed investigations are warranted to design novel combinational or sequential therapeutic approaches to enhance the efficacy of immunotherapy.

Zhao et al. described using immune checkpoint blockade (ICB) against ocular melanoma in a systemic review of the literature, including 16 articles containing data from ~850 melanoma patients. The authors concluded that ICB is an effective and safe treatment option against ocular melanoma. However, a detailed analysis of larger cohorts is needed since ICB is partially effective against ocular melanoma, with a significantly lower response rate than cutaneous melanoma. Since cutaneous melanoma and ocular melanoma are genetically and biologically distinct, it is of great interest to examine the correlations of genetic and transcriptomic profiles of the responders to ICB in both types of melanomas.

A severe side effect and safety concern of immune checkpoint inhibitors was described by Khimani et al. in a case report where in response to nivolumab and ipilimumab, a uveal melanoma patient developed Neuromyelitis Optica Spectrum Disorder. Though this is the only 3^rd^ such case in melanoma and the first in uveal melanoma, further analyses of more such cases are essential to completely understand the pathobiology of this disorder in response to the immune checkpoint inhibitors.

Approximately 80% of mutated melanomas are *BRAFV600E* mutants. Despite targeted BRAFi or BRAFi+MEKi being quite successful, the resistance against such targeted approaches is imminent. Misek et al. opportunistically investigated the sensitivity of “BRAFi resistant” melanomas to the mitotic inhibitors. This research article describes a unique second-line approach to identifying the mitotic inhibitors that can limit the progression of BRAFi-resistant melanoma. In a chemical screen on BRAFi sensitive and resistant cells, the authors identified compounds that disrupt mitosis, very specifically in the resistant cells. An interesting mechanism suggested by authors is cyclin B1 hyperactivity in resistant cells, which, unlike in non-resistant and normal cells, remains unaffected by the mitotic inhibitors.

To counter the resistance to targeted and immunotherapy,  Quaresmini et al. presented a case of nodular melanoma of the scalp, which was treated with electrochemotherapy (ECT). This manuscript describes the treatment regime and final prognosis of the patient. Interestingly, even after failing the first ECT, the second ECT induced the anti-tumor response leading to almost complete remission and a better prognosis where the patient is still living healthy. This case report highlighted the possibility of non-classical treatment regimes against melanoma which needs to be explored further in detail.

The rapid development of resistance against universal targeted therapies and immunotherapies has been a huge clinical challenge. In this regard, Zeng et al. reviewed the perspectives of personalized therapy against melanoma. The authors discussed the genetic variations among patients and their roles in designing individualized melanoma therapy against advanced and highly metastatic tumors.

Overall, the compiled studies in this issue range from pure research to clinical studies and appropriately fit into a uniform theme of “Mechanisms of resistance to the targeted therapy and immunotherapy in cutaneous melanoma”.

## Author contributions

SP wrote the draft, YQ and NA edited and finalized it with substantial intellectual inputs. All the authors approve this editorial for publication.

## Acknowledgments

SP acknowledges the startup support from Moffitt Cancer Cancer Center & Research Institute. NA acknowledges the Dr. Frederick E. Mohs Skin Cancer Research Chair endowment.

## Conflict of interest

The authors declare that the research was conducted in the absence of any commercial or financial relationships that could be construed as a potential conflict of interest.

## Publisher’s note

All claims expressed in this article are solely those of the authors and do not necessarily represent those of their affiliated organizations, or those of the publisher, the editors and the reviewers. Any product that may be evaluated in this article, or claim that may be made by its manufacturer, is not guaranteed or endorsed by the publisher.
